# Determination of Indolepropionic Acid and Related Indoles in Plasma, Plasma Ultrafiltrate, and Saliva

**DOI:** 10.3390/metabo13050602

**Published:** 2023-04-27

**Authors:** George M. Anderson

**Affiliations:** Department of Laboratory Medicine, The Child Study Center, Yale University School of Medicine, 230 S. Frontage Rd., New Haven, CT 06519, USA; george.anderson@yale.edu

**Keywords:** tryptophan, indolepropionic acid, indolelactic acid, indoleacetic acid, indoxylsulfate, indole, plasma ultrafiltrate, saliva, gut metabolome

## Abstract

The microbial metabolite indolepropionic acid (IPA) and related indolic metabolites, including indolecarboxylic acid (ICA), indolelactic acid (ILA), indoleacetic acid (IAA), indolebutyric acid (IBA), indoxylsulfate (ISO4), and indole, were determined in human plasma, plasma ultrafiltrate (UF), and saliva. The compounds were separated on a 150 × 3 mm column of 3 μm Hypersil C18 eluted with a mobile phase of 80% pH 5 0.01 M sodium acetate containing 1.0 g/L of tert-butylammonium chloride/20% acetonitrile and then detected fluorometrically. Levels of IPA in human plasma UF and of ILA in saliva are reported for the first time. The determination of IPA in plasma UF enables the first report of free plasma IPA, the presumed physiologically active pool of this important microbial metabolite of tryptophan. Plasma and salivary ICA and IBA were not detected, consistent with the absence of any prior reported values. Observed levels or limits of detection for other indolic metabolites usefully supplement limited prior reports.

## 1. Introduction

The microbial metabolites of tryptophan have become of greater interest due to an increasing recognition of their importance in human health and disease [1,2,3,4,5,6,7,8,9,10,11,12,13]. It appears that the most predominant indolic microbial metabolites in humans and rodents are indolepropionic acid (IPA), indolelactic acid (ILA), indoleacetic acid (IAA), and indole [14]. The latter compound is rapidly converted to 3-hydroxyindole (indoxyl) and subsequently to indoxylsulfate (ISO4). These metabolites, along with indolecarboxylic acid (ICA) and indolebutyric acid (IBA), have the potential to serve as bacterial signaling molecules and affect the composition and physiology of the gut microbiome [15]. A number of the indolic metabolites appear to influence host immune responses and gut permeability through actions at the aryl hydrocarbon receptor (AHR) and the pregnane X receptor (PXR) [9,16].

While all of the indolic metabolites are of potential importance, IPA is of particular interest [17]. IPA was first reported to be a bacterial metabolite of tryptophan in 1903 [18,19]. Subsequently, IPA has been found to be produced both by the deamination of tryptophan and by more complex routes that all preserve the indole moiety [9,20,21,22]. Research on IPA’s antioxidant and radical scavenging properties has steadily increased over the past 10–20 years. The research indicates that the compound has an important role to play in cytoprotection [23,24,25,26] and the immune response [27]. Recent studies have reported that lower plasma IPA levels are associated with human kidney disease [28,29], artherosclerosis [30], and diabetes [31,32]. Higher levels have been associated with diets high in fiber [33], while lower levels in human plasma have been associated with dietary meat [34]. The associations with fiber and meat consumption are consistent with an earlier report of very low levels of IPA in the feces of rats on a high-meat diet [35].

We report here on the measurement of IPA, along with related indoles, in human plasma, plasma ultrafiltrate (UF), and saliva. The determination of IPA in human plasma ultrafiltrate (UF) constitutes the first reported measurements of IPA in this sample type of any species. As will be discussed, the determination of free plasma IPA (and other indoles) might be of particular importance, given that for most compounds, free plasma levels are much more closely related to tissue levels than total plasma levels. More generally, the method we have developed has the potential to determine simultaneously all of the major indolic microbial metabolites of tryptophan with minimal sample preparation, high recoveries, high selectivity, and low detection limits. Prior reported methods have determined only a limited number of the indolic metabolites and have often involved more elaborate sample preparation.

## 2. Methods

Reagents/Chemicals: Indolepropionic acid (IPA), indolecarboxylic acid (ICA), indolelactic acid (ILA), indoleacetic acid (IAA), 1-methylindoleacetic acid (1-MeIAA), 2-methylindoleacetic acid (2-MeIAA), indolebutyric acid (IBA), indoxylsulfate (ISO4), indole (IND), sodium L-ascorbate, sodium acetate, glacial acetic acid, tetrabutylammonium bromide (Sigma-Aldrich), and HPLC grade water (J.T. Baker), acetonitrile and methanol (EMD Millipore), were all obtained from the listed suppliers.

Materials and Instrumentation: Ultrafiltration experiments were performed using 3 kDa cutoff Vivaspin 500 devices (GE Healthcare product # GE28-9322-18). HPLC was performed using a Hitachi L7250 auto-injector, Shimadzu LC-10AD pumps, a Thermo-Fisher 80 × 3.2 mm Hypersil column of 3 μm C18 (P/N 30103-153030), and a Shimadzu RF-20Axs fluorescence detector.

Chromatographic conditions: The mobile phase of 80% pH 5.0 0.01 M sodium acetate containing 1.00 g/L of tetrabutylammonium bromide/20% acetonitrile was delivered at a flow rate of 0.6 mL/min with an oven temperature of 40 °C. Compounds were detected fluorometrically with excitation and emission wavelengths of 285 nm and 355 nm, respectively. 

Plasma and Saliva Samples: Pooled and individual healthy volunteer human plasma (K2EDTA anticoagulated) samples and saliva samples were obtained from Innovate Research Inc. (Novi, MI, USA) and from the Yale Center for Clinical Investigation (YCCI) Biorepository. All samples were collected between the hours of 9 a.m. and 5 p.m.

All biospecimens were completely anonymous and were provided to the investigator without any identifying information or subject contact. The research was determined to not be “Human Research” according to Yale University Internal Review Boards’ form HRP-310 (version 12 December 2019) and as defined therein by the U.S. Department of Health and Human Services (DHHS) and by the U.S. Food and Drug Administration (FDA). 

Sample Preparation: The internal standard 1-MeIAA was added (10 ng per 100 μL of plasma; 1.0 ng per 100 μL of saliva), and proteins were then precipitated by the addition of 400 μL of ethanol (EtOH). After being vortex mixed for 2–3 s, samples were placed on ice for 5 to 10 min and then centrifuged at ~10,000× *g* for 5 min. The supernate was transferred to another tube, and 4:1 vol/vol of mobile phase containing no acetonitrile was added. Plasma ultrafiltrate (UF) was prepared by placing 200 μL plasma samples in 3 kDa cutoff Vivaspin 500 ultrafiltration units. Samples were incubated at 37 °C for 15 min and then centrifuged for 10 min at 10,000× *g*. Internal standard (1-MeIAA) was then added (10 μL of 0.10 ng/μL 1-MeIAA per 100 μL of UF), and the ultrafiltrate samples were directly injected. Recoveries of the compounds through the ultrafiltration process were tested by filtering 200 μL of standards (20 ng) prepared in phosphate-buffered saline.

Analyte concentrations were determined using an internal standard (I.S.) calculation: (sample analyte peak height/sample I.S. peak height) × Response Factor × concentration of I.S. added, where the intra-assay Response Factor (equal to I.S. standard peak height/analyte standard peak height) was determined for each assay.

## 3. Results

Recoveries: The results of standard addition studies for the nine compounds in human plasma and saliva (n = 3 separate preparations for each sample type) are presented in Table 1. The observed retention times, relative fluorometric responses, and limits of detection (LODs) are also given in Table 1. Standard curves for all analytes were linear (r > 0.99) to the highest value added in the standard addition studies. This was substantially greater than any of the observed endogenous concentrations (see Table 1). The mean recoveries were consistently high, with absolute recoveries ranging from 93.8% (ICA) to 117.8% (IND). All analytes were baseline separated from potential endogenous interferents. Intra- and inter-assay coefficients of variation (CVs) for the retention times of the analytes were less than 1% and 2%, respectively. 

Mean (±SD, n = 3) percent recoveries of the compounds through the ultrafiltration process were typically close to quantitative: ICA, 97.1 ± 1.5; IAA, 98.2 ± 0.6; ILA, 111.0 ± 0.7; 2MIA, 94.5 ± 2.7; IPA,95.7 ± 3.5; 1MIA, 95.4 ± 2.2; IBA, 91.4 ± 1.7. However, a lower recovery of 80.9 ± 2.8% was observed for ISO4, and a very low and variable recovery was seen for IND (8.4 ± 5.8%). Concentrations of free ISO4 were corrected for recovery; free plasma IND levels were not reported, given the low and variable recoveries.

Analyte Identities and Detection Limits: Identities of endogenous peaks were established based on retention times and concentrations observed after chromatography in alternative mobile phases with either differing pH (4.5), differing sodium acetate concentration (0.05 M), or with different organic modifier (methanol). The mean concentrations and limits of detection observed for the compounds in human plasma (total and free) and saliva are given in Table 2. All compounds detected were determined with within-assay and assay-to-assay coefficients of variance of less than 10% (n ≥ 3 for all CVs).

## 4. Discussion

### 4.1. Comparison to Prior Reports

The concentrations found for each of the compounds are given in Table 2, along with prior reported values. As noted, the method allowed the first determination of free plasma IPA and of salivary ILA, as well as the first estimation of the %-free IPA in plasma.

In general, the observed concentrations are in good agreement with prior reports. This was true for the total plasma values seen for IAA, ILA, and IPA, for free plasma IAA and ISO4, and for salivary ISO4 and IND. In addition, the low upper limits established here for total and free plasma ICA and IBA are consistent with an absence of prior reports of the compounds in plasma. However, a few substantial discrepancies were noted; these discrepancies, along with several specific compound and matrix issues, are discussed below.

Total Plasma ISO4: The Human Metabolome Data Base (HMDB) and several reviews cite the work of Ujhelyi et al. [44] when stating that mean total plasma levels of ISO4 in healthy controls are 535 ± 290 ng/mL. However, Ujhelyi and colleagues were actually reporting on free ISO4 concentrations observed for hemodialysis patients. Our observed mean of 507 ± 180 ng/mL is consistent with the total value reported by Gryp and colleagues of 595 ± 576 ng/mL [36] and not dissimilar to the two other reports [37,38] (see Table 2).

Total Plasma IND: The observed mean concentration for plasma IND of 3.05 ± 3.07 ng/mL was consistent with the summary mean of four previous reports (4.51 + 3.5 ng/mL) [14]. It can be pointed out that IND is volatile and can be lost during sample preparation, and this may have contributed to the large variance observed within and across studies.

Plasma Free IAA: Our observed mean plasma UF IAA concentration of 21.6 ± 15.8 ng/mL is in excellent agreement with the value reported by Morita and colleagues [40]. In vitro protein binding studies have reported a range of values for IAA binding to human serum albumin [45,46,47,48,49]. Our observed mean %-free value of 9.23 ± 2.45%, while about half of that seen by Morita and colleagues [39] of 18%, is in the midrange of the prior in vitro estimations. 

Plasma ILA: Our observed mean free plasma value for ILA of 1.34 ± 0.46 ng/mL and the calculated %-free value of 1.10 ± 0.63% are both substantially lower than those reported by Morita et al. [39] of 22.6 ± 16.4 ng/mL and 18%.

Salivary IAA: The two reported [41,42] mean concentrations for salivary IAA are 26.3 ± 83.7 ng/mL and 550 ± 554 ng/mL. Although both studies used LC-MS/MS methods to analyze salivary IAA concentration, the divergence is remarkable. Our observed mean of 236 ± 287 ng/mL (median 94 ng/mL) is intermediate to the prior reports and consistent with both reports in showing high inter-individual variation. 

Salivary IND: The one prior study of IND in human saliva reported mean levels of 40 ± 90 ng/mL using a GC-MS method [43]. Although we observed a substantially higher mean value (160 ± 269 ng/mL), our observed median of 26.2 ng/mL is consistent with the study of Cooke and colleagues [43]. We note that the mean value of 4.9 ± 4.1 ng/mL observed for the indole metabolite, ISO4, in saliva was in good agreement with two prior reports [38,40] (see Table 2).

### 4.2. General Discussion

The measurement of free plasma IPA, the presumed physiologically available form of IPA, provides an improved basis for examining the role of IPA in human health and disease. The free plasma concentration of IPA was very low, and the %-free value of 0.26% was even lower than expected, given previous in vitro estimations of 92 to 99% protein binding of IPA to human serum albumin [45,46,47]. The determination of free plasma has the potential to provide a measure that is more closely related to the effects and mechanisms of action of IPA than the measurement of total plasma IPA. Given the large protein-bound pool of IPA and the small free fraction, factors affecting the protein binding of IPA are probably critical in determining tissue exposure to IPA and the magnitude of IPA’s physiological effects. 

The high absorbances and quantum efficiencies of the indolic compounds led to high selectivity (few non-analyte peaks) and low limits of detection. Although LC-mass spectrometric methods are theoretically capable of determining the indoles with similar levels of detection, to date, no other method of any kind has been published for the determination of free plasma IPA.

The inter-individual variances observed for several of the compounds probably reflect the influence of multiple factors on the plasma and salivary concentrations. In addition to plasma protein binding, possible relevant factors include individual differences in diet, gut microbiome, and liver catabolism. Although multi-determined, the variations seen are promising in terms of being able to identify determinants of individual differences and to establish associations and correlates of human health and disease. 

The possibility the oral microbiome might contribute to salivary levels of the indole acids cannot be ruled out. It is relevant that salivary IND has been reported to arise mainly from production by oral bacteria [43]. Simultaneous determination of free plasma and saliva concentrations would be necessary to address these issues.

The method provides opportunities to replicate and extend prior studies that have associated some of the compounds with specific diseases and with certain aspects of diet and gut microbiome speciation and to examine the intra-lumen and systemic effects of the indolic metabolites. Investigations of healthy controls looking at the effects of sex and age, including early ontogeny, diet, and diurnal and seasonal variation, appear warranted. In general, the simultaneous measurement of the compounds across sample types offers advantages in these various kinds of clinical studies. 

Two limitations of the present study are the relatively low number of healthy control individuals examined and the absence of simultaneously obtained plasma and saliva samples. The restriction of the study to seven metabolites can be considered a limitation; however, we believe these compounds include most of the more important and abundant microbial indolic metabolites of tryptophan.

## Figures and Tables

**Table 1:** Percentage (%) recovery and chromatographic data.

COMPOUND	PLASMA	PLASMA	SALIVA	SALIVA	R_T_ (min)	RELATIVE RESPONSE *	LOD ** (pg)
ng/mL ADDED	400	40	40	10	---	---	---
ICA	93.8 ± 1.4	98.9 ± 3.4	107.2 ± 4.3	99.2 ± 5.4	6.4	0.091	2.0
IAA	96.2 ± 1.9	94.6 ± 2.6	96.9 ± 3.2	96.2 ± 4.8	7.6	1.04	0.2
ILA	105.8 ± 1.1	105.4 ± 2.9	109.3 ± 3.5	104.2 ± 6.2	8.6	0.43	0.4
2-MeIAA	99.3 ± 0.8	100.1 ± 1.4	108.2 ± 5.0	99.6 ± 7.0	9.6	0.47	0.4
IPA	101.7 ± 0.6	100.5 ± 3.1	101.3 ± 6.0	101.4 ± 6.3	14.0	0.64	0.3
1-MeIAA	99.3 ± 1.7	102.0 ± 4.3	105.5 ± 6.5	101.2 ± 6.3	16.6	1.0	0.2
ISO4	104.4 ± 1.0	104.5 ± 3.4	110.0 ± 4.9	102.7 ± 5.8	20.4	0.36	0.6
IND	101.0 ± 0.3	104.4 ± 4.1	101.4 ± 0.5	117.8 ± 6.2	22.2	0.35	0.6
IBA	99.0 ± 0.4	96.6 ± 3.5	91.5 ± 4.0	105.8 ± 5.9	24.4	0.19	1.0

* Response relative to the internal standard 1-methylindoleacetic acid (1-MeIAA) of equal amounts (1 ng) of analyte and 1-MeIAA observed under the standard conditions. ** Limits of detection (LOD, pg amount given a response twice the peak-to-peak noise) expressed as an absolute injected amount. Also equivalent to the ng/mL LOD when 25 μL of prepared total plasma or saliva samples were injected. Concentration LODs for free plasma were approximately 20-fold lower when 25 μL of ultrafiltrate was injected.

**Table 2:** Concentration (Mean ± SD) of indoles in human plasma, plasma ultrafiltrate (UF) and saliva.

**INDOLE**	**TOTAL PLASMA** **CONC. (n = 14) (ng/mL)**	**PRIOR REPORTED TOTAL PLASMA CONCENTRATIONS** **(ng/mL)**		
ICA	<2	No Prior		
IAA	225 ± 135	225 ± 50 summary mean ± SD [14]		
ILA	107 ± 20.3	205 ± 124 summary mean ± SD [14]		
IPA	142 ± 67.7	112 ± 23 summary mean ± SD [14]		
ISO4	507 ± 180	595 ± 576 [36], ~1030 [37], 1040 (median, IQR 650) [38]		
IND	3.05 ± 3.70	4.1 ± 3.5 summary mean ± SD [14]		
IBA	<1	No Prior		
**INDOLE**	**PLASMA UF CONC. (n = 14) (ng/mL)**	**PRIOR REPORTED** **PLASMA UF** **(ng/mL)**	**PLASMA** % FREE * (n = 14)	**PRIOR REPORTED** **%-FREE**
ICA	<0.1	No Prior	---	No Prior
IAA	21.6 ± 15.8	19.3 ± 14.0 [39]	9.23 ± 2.45	18% [39]
ILA	1.34 ± 0.46	22.6 ± 16.4 ng/mL [39]	1.10 ± 0.63	18% [39]
IPA	0.33 ± 0.12	No Prior	0.26 ± 0.13	No Prior
ISO4	12.3 ± 5.5	27.2 [40], 11 (IQR 16) [41]	2.56 ± 1.22	2.7% [40], 1% [41]
IND	NA	No Prior	---	No Prior
IBA	<0.05	No Prior	---	No Prior
**INDOLE**	**SALIVA CONC. (n = 7) (ng/mL)**	**SALIVA CONC. (n = 7)** **Median (IQR) (ng/mL)**		**PRIOR REPORTED SALIVA CONCENTRATIONS (ng/mL)**
ICA	<2	---		No Prior
IAA	236 ± 287	94 (390)		26.3 ± 83.7 [42], 550 ± 554 [41]
ILA	12.4 ± 15.2	7.6 (6.2)		No Prior
IPA	<1	---		No Prior
ISO4	4.9 ± 4.1	3.9 (5.4)		9.4 (IQR 7.6) [38], 8 (IQR 9) [40]
IND	160 ± 269	26.2 (197)		40 ± 90 [43]
IBA	<1	---		No Prior

* %Free values presented are mean (±SD) of 14 individually calculated %Free values: (100 × free plasma value/total plasma value).

## Data Availability

The data presented in this study are available on request from the author. Data is not publicly available due to privacy.
